# The rupture of atrioventricular groove after mitral valve replacement in an elderly patient

**DOI:** 10.1186/1749-8090-9-28

**Published:** 2014-02-08

**Authors:** Jin-Tae Kwon, Tae-Eun Jung, Dong-Hyup Lee

**Affiliations:** 1 Department of Thoracic and Cardiovascular Surgery, College of Medicine, Yeungnam University, 317-1 Daemyung 5 Dong, Namgu, Daegu, Korea

**Keywords:** Elderly, Mitral valve replacement, Rupture

## Abstract

Rupture of the left ventricle after mitral valve replacement, although infrequent, may be a highly lethal complication. This report describes the early diagnosis and successful repair of rupture of atrioventricular groove in an elderly patient who underwent mitral valve replacement.

## Background

As a result of demographic changes due to increase in average life expectancy, the age of patients undergoing cardiac surgery continues to rise. Consequently, elderly patients have more friable myocardium and more severe mitral annular calcification in mitral stenosis. Once it ruptures, treatment is difficult and results in a high mortality and morbidity for the patient. Therefore, left ventricular rupture following mitral valve replacement (MVR) should be diagnosed immediately and repaired without any hesitation.

## Case presentation

An 80-year old Korean female was referred with dyspnoea on exertion. She was New York Heart Association Class II. She did not have any prior medical history, and was diagnosed with moderate to severe mitral stenosis (valve area: 1.23 cm^2^ by 2D, mean diastolic pressure gradient, MDPG = 11.1 mmHg), moderate aortic stenosis (valve area: 1.21 cm^2^ by 2D, mean systolic pressure gradient, MSPG = 29.5 mmHg) and grade II tricuspid regurgitation. Preoperative echocardiography showed an enlarged left atrium (volume Index: 72 ml/m^2^) including thrombus (3.8 cm × 4.2 cm), 46 mm left ventricle end diastolic dimension (LVEDD), and 66% left ventricle ejection fraction. Her electrocardiogram showed atrial fibrillation, and her body surface area was 1.68 m^2^.

She underwent thrombectomy following MVR with a 29 mm Hancock II bioprosthesis (Medtronic, Minneapolis, MN, USA), aortic valve replacement with a 21 mm Hancock II bioprosthesis (Medtronic, Minneapolis, MN, USA) and tricuspid annuloplasty with the modified DeVega method. There were severe calcification of the both leaflets, commissures and part of posterial mitral annulus area. Therefore, we resected both leaflets. We did not preserve the subvalvular structures of the posterior leaflet.

**Figure 1:**
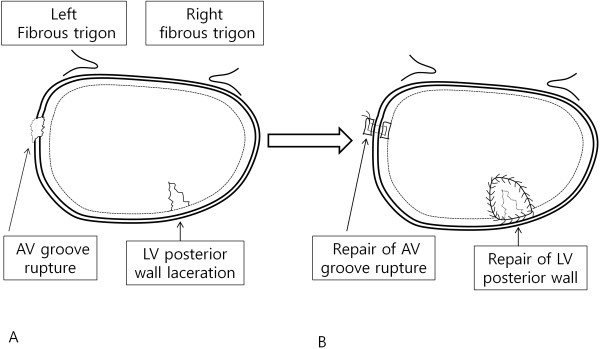
**Operative findings and procedures.** The small tear of atrioventricular groove and laceration of posterior left ventricular wall **(A)**. A simple bovine pericardial pledgetted suture was made at the small tear site of atrioventricular groove and bovine pericardial patch was sutured to the healthy endocardium of the left ventricular posterior wall **(B)**.

After the patient was taken off aortic cross clamp, we noticed extensive hematoma surrounding the posterior atrioventricular groove. We applied sutures from the outer surface of the heart, but haemorrhage continued at a different point of epicardium. We decided upon resumption of cardiopulmonary bypass (CPB). The left atrium was reopened and the mitral bioprosthesis was removed. The posterior atrioventricular junction and the posterior left ventricular (LV) wall were explored thoroughly. We found the site of the small tear just below the anterolateral commissure and laceration at the endocardium of posterior LV wall (Figure 1A). The small tear in the endocardium allowed blood to enter the atrioventricular groove. We repaired the site of small tear at the posterior atrioventricular groove with a small piece of bovine pericardial pledget and continuously sutured healthy endocardium with a bovine pericardial patch at the laceration area of LV posterior wall (Figure 1B). To avoid further injury to the LV posterior wall, a 25 mm ATS mechanical valve (ATS Medical, Minneapolis, MN, USA) was implanted again. Finally, a large bovine pericardium patch was placed over the outside oozing area of the anterior atrioventricular groove. We used blood cardioplegic solution which was infused every 20 minutes during the operation. The total pump time was 441 minutes and ACC time was 190 minutes.

The patient was taken into intensive care unit and extubated on the 5th postoperative day. She was discharged in a good condition after 30 days. Five months later, echocardiography showed an MDPG of 3.5 mmHg, MSPG of 12 mmHg, well-functioning prosthetic valves and no sign of thrombosis.

### Discussion

As a result of demographic changes due to increase in average life expectancy, the age of patients undergoing cardiac surgery continues to rise. For instance, the age describing elderly patients has risen from 65 years or older [1] to over 80 years in just 30 years. Consequently, the amount of degenerative valve diseases has increased due to the aging population. Likewise, improvements in operative techniques and postoperative care have contributed to an increase in elderly cardiosurgical patients.

Regardless of age, the incidence of LV rupture after MVR is approximately 1.2% and has a mortality rate of up to 75% [2]. Still, elderly patients have more friable myocardium and more severe mitral annular calcification in mitral stenosis. Older age, female gender, haemodialysis, and LVEDD < 50 mm are significant risk factors for LV rupture after MVR [3]. Once it ruptures, treatment is difficult and results in a high mortality and morbidity for the patient. Therefore, LV rupture following MVR should be diagnosed immediately and repaired without any hesitation.

Ruptures are classified into three types according to the location of the tear site [4,5]. Type I rupture is located in the atrioventricular groove, type II is at the base of the posterior papillary muscle, and type III occurs midway between type I and II sites. Each type can be further divided into intraoperative (early) and postoperative (delayed) rupture. Type I is the most common type and can be seen in the heavily calcified mitral valve annulus [6]. In Type I rupture, the pathologic finding is the separation of the annulus from the fibrous skeleton of the heart with the extravasations of blood into the myocardium and ultimately frank perforation and rupture. The outcome of type I rupture is worse than type II. This high mortality is because of the anatomical location of rupture requiring specific surgical approach and nearby course of the left circumflex coronary artery, which can be sutured and produce myocardial infarction.

Most ruptures result from surgical technique during the MVR or by stretch injury as a result of the untethering of LV through removal of the posterior leaflet of the mitral valve [4]. Preservation of the basal chordae of the posterior leaflet is important for the prevention of LV rupture [3]. Technical considerations such as excess excision of the mitral valve or papillary muscle, and excess traction have been reported, and these are usually associated with type I or II rupture [4,5]. In our procedure, we did not carefully make a stitch at the anterolateral commissure area and did not preserve the subvalvular structures of the posterior leaflet.

Several surgical methods for repair have been described in the literature, including both internal and external approaches. A report demonstrated that an off–pump biological tissue repair of the LV rupture was possible even in the presence of a large defective area [7].

We successfully repaired the rupture of the atrioventricular groove after MVR through early diagnosis, resumption of CPB, proper exposure and complete repair of the tear through an internal approach. Internal repair is considered the safest and most successful approach even though the implanted prosthesis has to be removed during repair [8]. However, prevention is the most important factor and by taking special care of elderly patients with calcified mitral annulus, the chances of LV rupture can be reduced.

## Conclusions

In actuality, LV rupture following the MVR is encountered more often than reported. And with careful precaution, the incidence can be minimized in elderly patients with calcified mitral annulus. Also, LV rupture can be repaired promptly following precise localization with low mortality.

## Consent

Written informed consent was obtained from the patient for publication of this case report and accompanying images. A copy of the written consent is available for review by the Editor-in-Chief of this journal.

## Abbreviations

AV: Atrioventricular; LV: Left ventricle; MVR: Mitral valve replacement; LVEDD: Left ventricle end diastolic dimension; CPB: Cardiopulmonary bypass; MDPG: Mean diastolic pressure gradient; MSPG: Mean systolic pressure gradient.

## Competing interests

The authors declare that they have no competing interests.

## Authors’ contributions

JK and TJ wrote the draft of the manuscript and obtained the written consent. DL performed the literature review and participated in the manuscript writing and helped to the final writing of the paper and gave final approval of the manuscript. All authors have read and approved the final manuscript.
